# Inhibition of PTCH1 drug efflux activity enhances chemotherapy efficacy against triple negative breast cancers

**DOI:** 10.1016/j.tranon.2026.102777

**Published:** 2026-04-18

**Authors:** Sarah Cogoluegnes, Sandra Kovachka, Thierry Dubois, Roberto Würth, Elisa Donato, Andreas Trumpp, Michel Franco, Frédéric Luton, Stéphane Azoulay, Isabelle Mus-Veteau

**Affiliations:** a University Côte d’Azur, CNRS, INSERM, IPMC, 660 route des Lucioles, Valbonne 06560, France; b University Côte d’Azur, CNRS, ICN, 28 Avenue Valrose, 06108, Nice CEDEX 2, France; c Institut Curie, PSL University, Sorbonne Université, Translational Research Department, CNRS UMR144 Cell Biology and Cancer, Breast Cancer Biology Group, 26 rue d’Ulm, Paris 75005, France; d Division of Stem Cells and Cancer, German Cancer Research Center (DKFZ) and DKFZ-ZMBH Alliance, Heidelberg 69120, Germany; e Heidelberg Institute for Stem Cell Technology and Experimental Medicine (HI-STEM gGmbH), Heidelberg 69120, Germany; f German Cancer Consortium (DKTK), Heidelberg, Germany

**Keywords:** TNBC, PTCH1, Drug efflux, Chemotherapy resistance, Doxorubicin, Docetaxel

## Abstract

Our previous work has revealed that PTCH1, a Hedgehog receptor which is expressed in many aggressive cancers, plays a role in transporting chemotherapeutic drugs out of cancer cells and contributes to their resistance to chemotherapy. PTCH1 transports drugs via the proton motive force using the 'reverse pH gradient', which makes its drug efflux activity specific to cancer cells and PTCH1 a highly relevant and specific new therapeutic target for cancer treatment. We have identified a small molecule produced by a marine sponge as being able to inhibit PTCH1 efflux activity and increase the efficacy of vemurafenib treatment on BRAF-mutated melanoma cells in vitro and in vivo in mice. In the present study, we demonstrate the presence of *PTCH1* mRNA in tumour samples and circulating tumour cells from breast cancer patients, particularly those with triple-negative breast cancer (TNBC). TNBC is the most aggressive breast cancer subtype, characterised by a high risk of resistance to chemotherapy and a high potential for relapse. Our analyses reveal that high levels of *PTCH1* mRNA are associated with a poor prognosis in TNBC patients. We found that inhibiting PTCH1 drug efflux activity significantly increased the cytotoxic effect of chemotherapies such as doxorubicin and docetaxel in three TNBC cell lines. Overall, our findings suggest that PTCH1 plays a role in the resistance of TNBC cells to chemotherapy, and that using a PTCH1 efflux inhibitor during neoadjuvant or adjuvant therapy could enhance the efficacy of treatment against PTCH1-expressing TNBC, while preventing treatment resistance, relapse, and metastasis formation.

## Highlights

• PTCH1 is expressed in tumour samples from breast cancer patients.
• High PTCH1 expression is associated with a poor prognosis.
• PTCH1 is a drug efflux pump involved in breast cancer resistance to treatment.
• PTCH1 inhibition increases the efficacy of chemotherapy on breast cancer cell lines.
• High therapeutic potential of targeting PTCH1 to prevent breast cancer relapse.

## Graphical abstract

**Figure:**
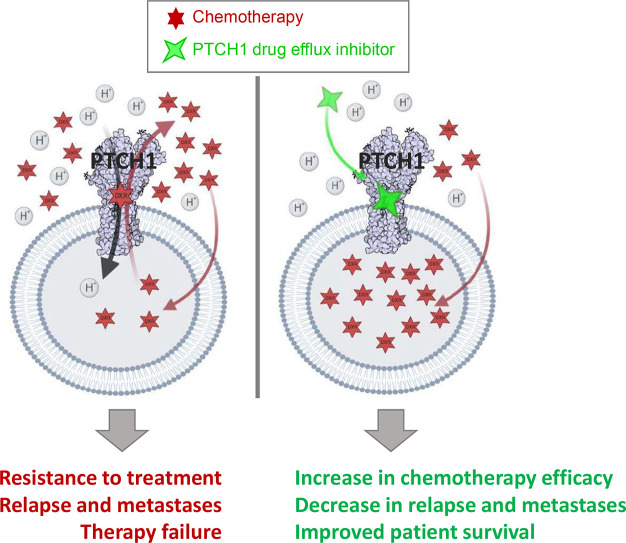

## Introduction

Breast cancer (BC) is the most prevalent tumour and the leading cause of cancer-related deaths among women worldwide, with approximately 2.2 million new cases and 685,000 deaths reported globally in 2020 [1,2]. It encompasses a complex spectrum of diseases, with various genetic and molecular features contributing to tumour development and progression [3,4]. Invasive breast cancers (IBCs) are classified using histological evaluation, molecular profiling, and surrogate markers. The World Health Organization (WHO) recognises 18 distinct histological subtypes of IBC. The most common of these is invasive carcinoma of no special type (NST), accounting for 40% to 80% of cases [5]. IBC can be subdivided into molecular subtypes: HER2-enriched, luminal A, luminal B and basal-like, based on tumour gene expression patterns. In clinical practice, surrogate markers such as oestrogen receptor (ER), progesterone receptor (PR) and HER2, ascertained via immunohistochemistry (IHC), are frequently employed for tumour classification [6]. 15–20% of breast cancers lack ER and PR expression and HER2 gene amplification and are classified as triple-negative breast cancer (TNBC). These subtypes exhibit distinct biological characteristics, clinical outcomes and treatment responses [7].

A variety of approaches have been employed in the treatment of breast cancer [8,9]. Chemotherapy, such as docetaxel, can be administered either before (neoadjuvant) or after (adjuvant) surgery and is tailored to the tumour's specific characteristics [10]. Endocrine therapies target oestrogen receptors (ERs), either by reducing oestrogen levels or by blocking oestrogen-induced stimulation of breast cancer cells [11]. However, around half of hormone receptor-positive breast cancers are resistant to this treatment [12]. Patients with TNBC do not benefit from established endocrine or HER2-targeted therapies due to the absence of relevant receptor markers, and general chemotherapy remains the standard treatment for non-surgical TNBC. Nevertheless, fewer than 30% of patients achieve a complete response, and both recurrence and mortality rates remain higher than for non-TNBC subtypes [13]. Although breast cancer (BC) survival has improved over the last few decades, treatment resistance still represents a significant unmet clinical need [3].

Similar to other developmental pathways, aberrant Hedgehog (HH) pathway activity plays a crucial role in the initiation and progression of various tumour types [14]. O'Toole et al. [15] observed that the expression of Hedgehog ligands, such as SHH, was significantly associated with an increased risk of metastasis and breast cancer-specific death, as well as a basal-like phenotype, in a cohort of patients with invasive ductal carcinoma of the breast. Their data suggested that epithelial–stromal HH signalling, driven by ligand expression in carcinoma cells, promotes breast cancer growth and metastasis. Jeng et al. [16] also observed in a cohort of patients with invasive breast carcinoma that, compared with paired non-cancerous tissue, higher expression of mRNA from HH pathway components (SHH, PTCH1, GLI1 and SMO) in breast cancer tissue correlates with invasiveness and is associated with an increased risk of recurrence. We discovered that the SHH receptor, PTCH1, acts as a multidrug transporter, refluxing chemotherapeutic agents out of cancer cells and contributing to the chemotherapy resistance of adrenocortical carcinoma and melanoma cells [[17], [18], [19], [20]]. We demonstrated that PTCH1 utilises the proton motive force to expel drugs in a manner similar to bacterial efflux pumps from the RND family [17]. Therefore, PTCH1 acts as a drug efflux pump only in cancer cells that exhibit an acidic extracellular pH due to their high glucose consumption, unlike normal cells that exhibit a slightly more basic extracellular pH than the intracellular pH [21]. Furthermore, PTCH1 is expressed at very low levels in adults. This makes PTCH1 a highly relevant therapeutic target, as its inhibition would affect only cancer cells, unlike the multidrug transporters from the ABC family, such as P-glycoprotein (P-gp), whose inhibition is toxic to healthy cells [22]. Screening approximately 2,000 small molecules enabled us to identify panicein A hydroquinone (PAH), extracted from the marine sponge Haliclona mucosa, as an inhibitor of PTCH1 drug efflux activity [23]. We synthesised PAH and demonstrated that it increased the efficacy of conventional chemotherapies, such as doxorubicin, and targeted therapies, such as vemurafenib (an inhibitor of the BRAF mutant V600E), on BRAF V600E-expressing melanoma cells, in both in vitro and in vivo models [20,24,25].

In the present study, we examined the expression levels of PTCH1 mRNA in tumour samples from several cohorts of breast cancer patients, in circulating tumour cells isolated from blood samples of patients with metastatic breast cancer and in various breast cancer cell lines. We observed that PTCH1 is expressed more strongly in TNBC. PTCH1 drug efflux inhibition was found to significantly reduce chemotherapy efflux in three TNBC cell lines, leading to a substantial increase in the efficacy of doxorubicin and docetaxel. Our data highlight the therapeutic potential of targeting PTCH1 drug efflux activity to improve treatment outcomes for TNBC patients.

## Materials and methods

### Chemical and biological material

Panicein A hydroquinone was synthesized as described in [20].

Doxorubicin hydrochloride and docetaxel were purchased from Sigma-Aldrich and Acros-Organics, respectively.

Human breast cancer cell lines MDA-MB-231, MDA-MB-468 and HCC-38 were purchased from ATCC. Cells were cultured at 37 °C in a 5% CO_2_ / 95% air water-saturated atmosphere in DMEM medium supplemented with 10% fetal bovine serum and penicillin/streptomycin for MDA-MB-231, in DMEM-F12 medium supplemented with 2 mM HEPES, 10% fetal bovine serum and penicillin/streptomycin for MDA-MB-468, and in RPMI medium supplemented with 1.5 g/L sodium bicarbonate, 10 mM HEPES, 1 mM sodium pyruvate, 10% fetal bovine serum and penicillin/streptomycin for HCC-38.

K699 *S. cerevisiae* yeast strain (Mata, ura3, and leu 2–3) and K699 S. c. strain expressing human PTCH1 were grown as described [26].

### PTCH1 mRNA expression analyses in human biopsies and in human breast cancer cell lines

TCGA cohort: the publicly available RNA-SeqV2 Level 3 dataset (January 2015) were downloaded from The Cancer Genome Atlas (TCGA) breast invasive carcinoma cohort (http://cancergenome.nih.gov/) [27] and integrated into a platform in knowledge data integration (KDI) at Institut Curie (https://bioinfo-portal.curie.fr). We classified the breast cancer subgroups, based on the immunohistochemical status for ER, PR and HER2, as described [28]. TNBC (ER-, PR-, HER2-), HER2 (ER-, PR-, HER2+), luminal A (ER+ and/or PR+, HER2-), and luminal B (ER+ and/or PR+, HER2+).

Cell lines: total RNA from the different breast cancer cell lines was extracted and hybridized to GeneChip™ Human Exon 1.0 ST arrays (Affymetrix) as previously described [29].

Using breast cancer gene-expression miner (bc-GenExMiner v4.5): this is a breast cancer-associated web portal (http://bcgenex.ico.unicancer.fr) with a statistical mining module allowing several differential gene expression analyses based on microarray or RNAseq transcriptomic data on sixty-two breast cancer cohorts [30]. Prognostic analyses were performed on this web portal using exhaustive prognostic analysis which permits to screen the prognostic impact of a gene or a specific Affymetrix® probeset ID on all possible combinations of population (significant results may be considered robust if more than 5 combinations among the 27 give a significant result. If there are only 5 combinations or less with a *p*-value < 0.05, one cannot exclude a false discovery problem) and intrinsic molecular subtypes prognostic analysis which permits to assess the prognostic impact of a gene or a specific Affymetrix® probeset ID within groups of patients with a certain intrinsic molecular subtypes as defined by Single Sample Predictors (SSP) and/or Subtype Clustering Models (SCM) (Supplementary Fig. 1).

### PTCH1 expression analysis on organoids derived from circulating tumor cells from breast cancer patients

For the analysis of *PTCH1* expression on CTC-derived organoids from metastatic breast cancer individuals, data from Würth and colleagues [31] were re-analyzed with DESeq2 package (v1.26.0) using RStudio (v1.4).

### PTCH1 knock-down

MDA-MB-231 cells were transfected with 60 nmol of human *PTCH1* Silencer® Select Pre-designed siRNA (Ambion, #4392420, s11441 (sense: 5’GCACUUACUUUACGACCUAtt3’, as: 5’UAGGUCGUAAAGUAAGUGCtg3’) or control (medium GC) siRNA oligos (Invitrogen) using Lipofectamine RNAiMAX reagent (Invitrogen) following the manufacturer's protocol, then seeded in 24-well plates and incubated at 37 °C and 5% CO_2_ for 16 h before western blotting and doxorubicin fluorescence measurements as described in Signetti et al. [20].

### SDS-PAGE and western blotting

Total RIPA extracts from cells were prepared. Protein concentrations were determined by the Bradford Protein Assay (Bio-Rad). Samples (50 to 80 µg) were separated on SDS-PAGE and transferred to nitrocellulose membranes (Amersham) using standard techniques. After 1 h at room temperature in blocking buffer (20 mmol/L Tris-HCl pH 7.5, 45 mmol/L NaCl, 0.1% Tween-20, and 5% non-fat milk), nitrocellulose membranes were incubated overnight at 4 °C with the monoclonal rat anti-PTCH1 antibody from R&D system biotechne clone 413220 (1/1000), the monoclonal mouse anti-P-gp antibody from abcam (ab3366, 1/1000), the rabbit anti-ABCG2 antibody from GeneTex (GTX50793, 1/500) or the rabbit anti-GAPDH antibody from Elabscience (1/20000). After 3 washes, membranes were incubated for 45 min with anti-rat (1:10000), anti-rabbit (1:2000) or anti-mouse (1:5000) immunoglobulin coupled to horseradish peroxidase (Dako). Detection was carried out with an ECL Prime Western Blotting detection reagent (SuperSignal West Femto Maximum Sensitivity Substrate from ThermoScientific) on a Fusion FX imager (Vilber Lourmat), and analyses were performed using ImageJ software.

### Cytotoxicity assays

*On adherent cells*: Cells were seeded in 96-well plates in triplicate and grown in medium to achieve 70% to 80% confluence. Medium was then removed and replaced with 100 µL/well of complete medium containing PAH or DMSO as a control. After 15 min, 100 µL of complete medium containing serial dilutions of doxorubicin or docetaxel were added. Plates were incubated at 37 °C and 5% CO_2_. After 24 or 48 h, cells were incubated for 3 h at 37 °C with 100 µL/well neutral red (NR) solution (50 µg/mL in medium) following the manufacturer’s protocol. Measurements were made in microplate readers (Multiskan Go Microplate Spectrophotometer from Thermo Scientific). IC_50_ was defined as the concentration that resulted in a 50% decrease in the number of viable cells, and IC_50_ values were calculated using GraphPad Prism 6 software.

*On spheroids*: MDA-MB-231 cells were plated in ultralow attachment 24 well plates in complete medium containing PAH or DMSO as a control and 100, 200, 250 or 300 µM docetaxel. Plates were incubated at 37 °C and 5% CO_2_. After two weeks, pictures of each well were taken using the Cytation 5 cell imaging system from Biotek.

### Transwell invasion assay

The cell invasion assay was performed according to the manufacturer’s instructions (Costar) with a 24-well transwell plate containing transwell cell culture chamber inserts with polycarbonate membranes to study cell invasion. Briefly, 3 × 10^5^ cells per well were plated in the upper chamber of the transwell plate in complete culture medium containing 15 µM PAH or DMSO, and 0 or 1 µM doxorubicin in one plate and 0 or 20 µM docetaxel in the other one. Complete culture medium was added to the lower chamber, and transwell plates were incubated at 37 °C in 5% CO_2_ atmosphere 24 h for the one treated with doxorubicin or 48 h for that treated with docetaxel. Then, the upper chambers were removed, and the wells (lower chambers) were fixed, stained with crystal violet and observed on a microscope with X5 objective.

### Wound-healing assay

Once cells were confluent in 24-well plates, a wound was performed with a P200 pipette tip, and the medium was replaced by fresh medium containing or not 5 µM docetaxel or/and 5 µM PAH. Pictures of each well were taken immediately after wounding, and 72 h after wounding with a Leica DM IRB (5X objective). The width of the wound was measured using ImageJ software and reported as final wound width / initial wound width in percentage.

### Doxorubicin intracellular measurements

For doxorubicin accumulation measures, cells were seeded on coverslips in 24-well plates, treated or not with si-RNA, allowed to grow to 80% confluence, and incubated at 37 °C and 5% CO_2_ with 10 μM doxorubicin in physiological buffer (140 mM NaCl, 5 mM KCl, 1 mM CaCl_2_, 1 mM MgSO_4_, 5 mM glucose, 20 mM HEPES, pH 7.4) supplemented or not with 50 µM docetaxel. After 1 h, coverslips were immediately fixed with 4% PFA, rapidly washed with PBS and mounted in SlowFade Gold antifade reagent with DAPI (Invitrogen).

For doxorubicin efflux measures, cells were seeded on coverslips in 24-well plates, allowed to grow to 80% confluence, and incubated at 37 °C and 5% CO_2_ with 10 μM doxorubicin in physiological buffer. After 2 h, three coverslips were immediately fixed with 4% PFA for the doxorubicin loading control, rapidly washed with PBS and mounted in SlowFade Gold antifade reagent with DAPI (Invitrogen). The other coverslips (triplicate per condition) were incubated with physiological buffer supplemented with DMSO or 10 µM of PAH under gentle shaking at room temperature and protected from light. After 30 min, coverslips were fixed with 4% PFA, washed and mounted as described above.

Images were acquired with a Zeiss Axioplan 2 fluorescence microscope coupled to a digital charge-coupled device camera using a 40X/1.3 Plan NeoFluar objective and filters for Alexa 594. Doxorubicin fluorescence was quantified using ImageJ software. Sampling of cells was performed randomly. About 100 cells (from three wells) were scored per condition per experiment.

### Yeast growth inhibition by drugs

*S. cerevisiae* expressing wild-type human PTCH1 were pre-cultured minimum medium to OD_600nm_ = 2, diluted to OD_600nm_ = 0.2 in rich medium supplemented or not with 5 µM PAH and with 15 µM doxorubicin or 400 µM of docetaxel, and grown at 18 °C in 96-well plates (BD Biosciences). Absorbance at 600 nm was recorded during growth.

### Statistical analysis

All results represent at least three independent replications. Data are shown as mean value ± SEM. Prism 6 (GraphPad) was used to determine IC_50_ values and other statistical analyses using one-way analysis of variance (ANOVA) followed by Bonferroni’s Multiple Comparison Tests.

## Results

### PTCH1 mRNA is expressed in human breast cancer cells and circulating tumour cells

**Fig. 1:**
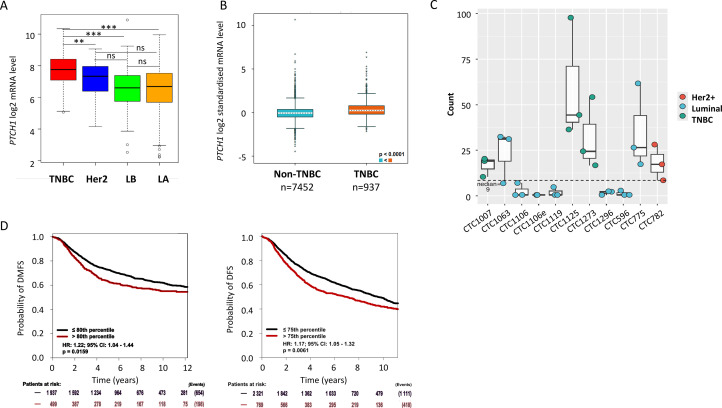
***Ptch1 is expressed in breast cancer patients.* A.** Relative *PTCH1* RNA expression in TNBC (red), Her2 (blue), luminal B (LB, green), and luminal A (LA, orange) in the TCGA cohort is illustrated by box plots (log_2_ transformed). Outliers are shown within each population (open circles). Student's t test was used to compare RNA levels between two groups. The *p* values are indicated (*P < 0.05; **P < 0.01; ***P < 0.001; ns *P* > 0.05). **B.** Normalized *PTCH1* mRNA expression according to TNBC (IHC) status from all DNA microarray data from bc-GenExMiner v4.5 illustrated by box plots (log_2_ transformed) [30]. **C**. Boxplots showing *PTCH1* mRNA expression (normalized counts from RNA seq data) in circulating tumor cells (CTC) isolated from metastasis breast cancer patient peripheral blood [31]. **D.** High *PTCH1* mRNA expression is associated with a poorer prognosis in breast cancer. Distant Metastasis Free Survival (DMFS) and disease-free survival (DFS) data based on *PTCH1* mRNA expression were obtained using the exhaustive prognostic analysis on all status breast cancers on the bc-GenExMiner v4.5 web portal, and illustrated by Kaplan–Meier (KM) curves for all ER and PR breast cancers with positive nodules. The obtained Hazard Ratio (HR) with 95% confidence interval and log-rank *p*-values are shown.

Analysis of The Cancer Genome Atlas (TCGA) cohort showed that PTCH1 mRNA is expressed in all breast cancer subgroups, with the highest expression observed in triple-negative breast cancer (TNBC) (Fig. 1A). Analysis of PTCH1 expression using the breast cancer gene-expression miner (BC-GenExMiner v4.5) [30] also revealed that PTCH1 mRNA is significantly more abundant in TNBC than in other breast cancer subtypes (Fig. 1B). Furthermore, we analysed PTCH1 expression in CTCs using a recent RNA-seq dataset of CTC-derived organoids (CDOs) isolated from 11 liquid biopsies of 10 patients with metastatic breast cancer, representing all major breast cancer subtypes [31]. PTCH1 expression varied among the CDOs, with null/low expression observed in five CDOs and higher expression in six CDOs. Notably, higher PTCH1 expression was observed in all (3/3) CDOs established from TNBC patients (Fig. 1C). These data indicate that PTCH1 is not only expressed in patient breast tumours, but also in-patient CTCs, which are well known to drive metastasis — the leading cause of death in individuals with breast cancer.

We then investigated the potential clinical significance of PTCH1 mRNA expression levels using prognostic analyses performed on the Breast Cancer Gene-Expression Miner web portal. This exhaustive analysis, which allows the prognostic impact of PTCH1 to be screened for all possible population combinations, revealed that high PTCH1 mRNA expression was associated with lower distant metastasis-free survival (DMFS) and disease-free survival (DFS) in 14 and seven combinations, respectively, but not with lower overall survival (OS). Fig. 1D illustrates this with Kaplan-Meier curves obtained from patients with all ER and PR statuses and node-positive BC. However, an analysis of the intrinsic molecular subtypes of patients with high proliferative ER+/HER2- BC revealed that high PTCH1 mRNA expression was associated with lower DMFS, DFS and OS (see Supplementary Fig. 1).

**Fig. 2:**
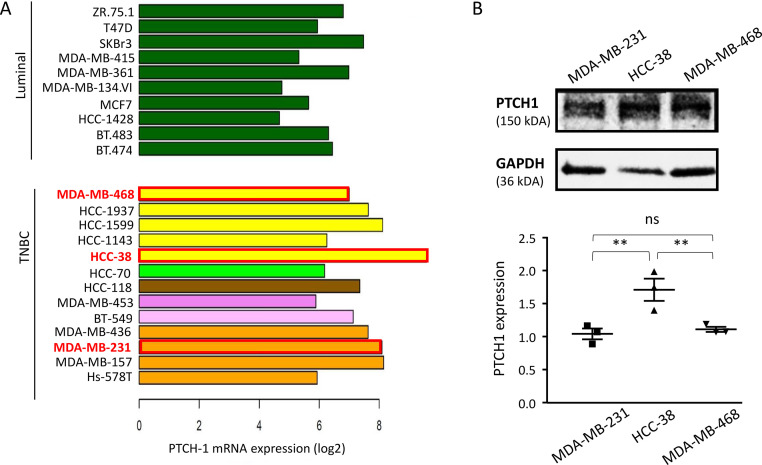
***Ptch1 is expressed in breast cancer cell lines.* A.***PTCH1* mRNA expression (log_2_ transformed) in various luminal and HER2 breast cancer cell lines (dark green) and in TNBC cell lines (other colors). TNBC cell lines are depicted according to the “Lehmann TNBC subtype” nomenclature [32]: basal-like 1 (yellow), basal-like 2 (pale green), immunomodulatory (brown), luminal androgen receptor (dark pink), mesenchymal (pale pink) and mesenchymal stem-like (pink). **B.** PTCH1 protein expression in three TNBC cell lines. Western-blot was performed on 50 µg extracts from TNBC cell lines (MDA-MB-231, HCC-38 and MDA-MB-468) with antibodies directed against PTCH1. PTCH1 and GAPDH signals were quantified using ImageJ software. Data presented are the mean ± SEM of at least 3 independent experiments. Significance is calculated using Oneway ANOVA Turkey’s multiple comparisons test and attained at *P* < 0.05 (*).

Transcriptomic analysis of 23 breast cancer cell lines revealed variable PTCH1 mRNA levels, with TNBC cell lines tending to exhibit the highest levels compared to other breast cancer cell lines (Fig. 2A). Western blot analysis confirmed PTCH1 protein expression in three TNBC cell lines (MDA-MB-231, HCC-38 and MDA-MB-468) and showed that HCC-38 cells express significantly higher levels of PTCH1 protein than MDA-MB-231 and MDA-MB-468 cells (Fig. 2B).

These studies confirm that PTCH1 is expressed in breast cancer, particularly in TNBC. If PTCH1 is indeed involved in TNBC's resistance to conventional chemotherapies such as doxorubicin and docetaxel, inhibiting it could increase the efficacy of these treatments.

### Docetaxel is a PTCH1 substrate

**Fig. 3:**
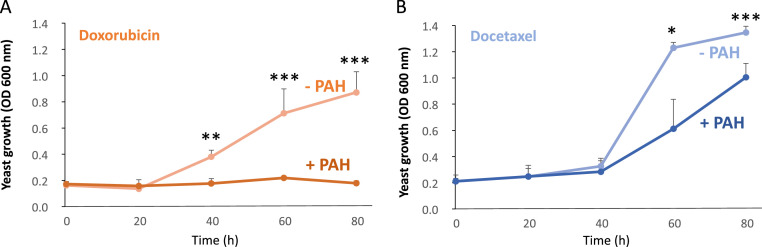
*PTCH1****confers to yeasts a resistance to doxorubicin and docetaxel which is inhibited by PAH***. Control yeasts and hPTCH1-expressing yeasts were grown in the presence of 15 µM doxorubicin (**A**) or 400µM docetaxel (**B**), supplemented or not with 5 µM of PAH. The growth of yeasts was measured by absorbance at 600 nm in function of time. The growth curves shown represent the average of at least three independent experiments with sem. Significance is calculated using Student test and attained at *P* < 0.05 (*).

The standard treatment for TNBC consists of chemotherapies such as platinum agents (cisplatin and carboplatin), taxanes (paclitaxel and docetaxel) and anthracyclines (doxorubicin and epirubicin) [33,34]. We previously demonstrated that PTCH1 can efflux doxorubicin using a *Saccharomyces cerevisiae* yeast strain that overexpresses human PTCH1. We observed that these yeasts were able to grow in the presence of a doxorubicin concentration that prevented the parental yeast strain from growing, and that they effluxed more doxorubicin than the parental yeast strain ([17], Fig. 3A). To determine whether PTCH1 can efflux docetaxel as well as doxorubicin, we cultivated yeasts overexpressing PTCH1 in the presence of docetaxel. Here, we show that expressing PTCH1 enables yeasts to grow in the presence of 400 µM of docetaxel (Fig. 3B), adding the inhibitor of PTCH1 drug efflux activity (PAH) to the medium inhibits the growth of yeasts expressing PTCH1 in the presence of docetaxel, as it does in the presence of doxorubicin (Fig. 3A). These results suggest that PTCH1 plays a role in the efflux of docetaxel and that targeting PTCH1 could be an effective strategy for increasing the efficacy of docetaxel in TNBC cells.

### Inhibiting PTCH1 drug efflux increases the sensitivity of TNBC cells to chemotherapies in both 2D and 3D cultures

**Fig. 4:**
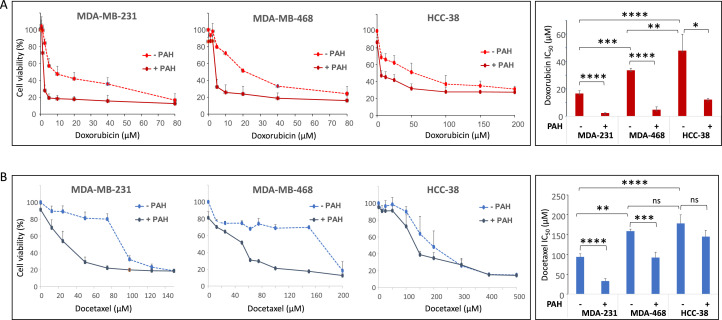
***PTCH1 drug efflux inhibitor PAH increases the sensitivity of TNBC cells to chemotherapy****.* Cell viability was measured after 24 h or 48 h treatment with increasing concentration of docetaxel or doxorubicin respectively on MDA-MB-231, MDA-MB-468 and HCC-38 cell lines in the absence or the presence of 15µM PAH. IC_50_ values (corresponding to the concentration of chemotherapy inducing 50% of cell death) were calculated. Data reported are the mean ± SEM of at least 3 independent experiments. Significance is attained at *P* < 0.05 (*).

We then tested the effects of doxorubicin and docetaxel on the viability of three TNBC cell lines with endogenous PTCH1 expression (MDA-MB-231, HCC-38 and MDA-MB-468), as shown in Fig. 2B. The cells were grown to 80% confluency and then treated with doxorubicin or docetaxel for 24 or 48 h, respectively, in the absence or presence of the PTCH1 drug efflux inhibitor PAH. As shown in Fig. 4, adding PAH to doxorubicin or docetaxel increased the cytotoxicity of both chemotherapies, significantly decreasing their IC₅₀ in 2D culture (see also Supplementary Table 1).

**Fig. 5:**
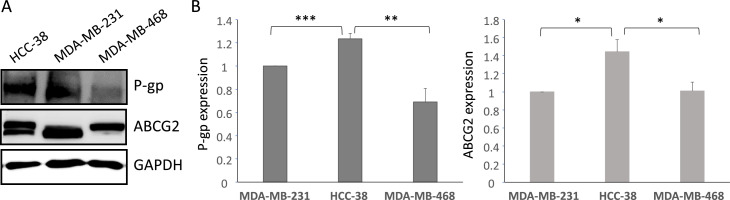
***Other multidrug transporters are expressed in TNBC cell lines.* A.** P-gp and ABCG2 protein expression in three TNBC cell lines. Western-blot was performed on 50 µg extracts from each TNBC cell line (MDA-MB-231, HCC-38 and MDA-MB-468) with antibodies directed against P-gp or ABCG2 and GAPDH. **B.** P-gp, ABCG2 and GAPDH signals were quantified using ImageJ software. Data presented are the mean ± SEM of at least 3 independent experiments. Significance is calculated using Oneway ANOVA Turkey’s multiple comparisons test and attained at *P* < 0.05 (*). **P < 0.01; ***P < 0.001; ns *P* > 0.05.

However, PAH had a less pronounced effect on HCC-38 cells, despite these cells expressing the highest amount of PTCH1. Notably, HCC-38 cells were found to express significantly higher levels of P-glycoprotein (P-gp) and ABCG2 than MDA-MB-231 and MDA-MB-468 cells (Fig. 5). P-gp and ABCG2 are multidrug transporters from the ABC family that are well known to contribute to the drug resistance of cancer cells. This could explain why PAH has a lesser effect on HCC-38 cells than on MDA-MB-231 and MDA-MB-468 cells.

**Fig. 6:**
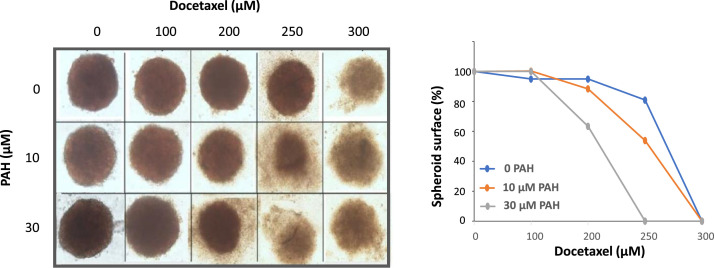
***PAH increases docetaxel efficacy against TNBC spheroids.*** MDA-MB-231 cells were plated in ultra-low attachment surface 24 well plates in complete medium and treated with increasing concentrations of docetaxel in the presence of DMSO (control, 0 PAH), PAH 10 µM or 30 µM. After 2 weeks pictures were taken using Cytation 5 cell imaging system from Biotek. The surface of spheroids was calculated and reported after normalization on the condition without docetaxel for each concentration of PAH.

We then tested the effect of PTCH1 drug efflux inhibition on MDA-MB-231 spheroids, as these more closely resemble tumours than 2D cultures. We observed that the addition of 10 or 30 µM PAH destroyed the spheroids at 250 µM and 200 µM docetaxel, respectively; while concentrations of docetaxel alone had no effect (Fig. 6). This experiment confirmed that PTCH1 drug efflux inhibition significantly enhances the cytotoxic effects of chemotherapy on TNBC cells.

### PTCH1 drug efflux inhibition enhances the effect of chemotherapies on TNBC cells migration

**Fig. 7:**
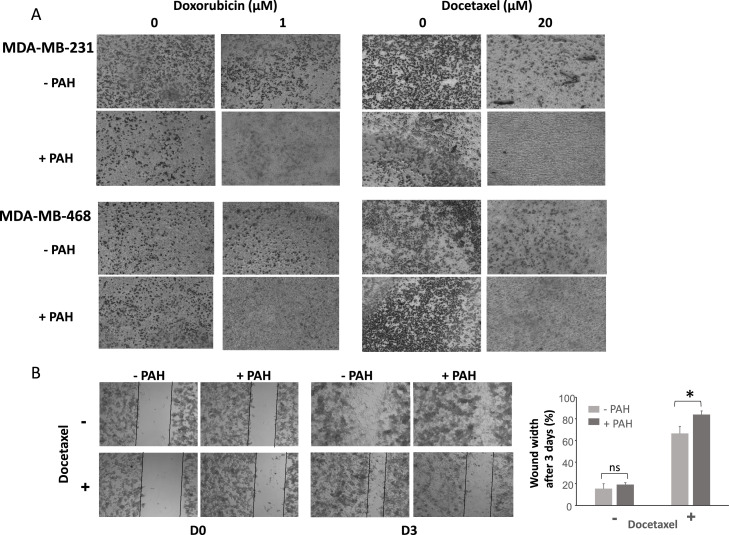
***PAH addition to chemotherapy inhibits migration of TNBC cells. A.*** 100000 cells were seeded on membrane from Transwell plates. Cells were treated 24 h with doxorubicin ± 15µM PAH or 48 h with docetaxel ± 15µM PAH. After fixation and staining with crystal violet, wells were observed on a microscope with X5 objective. ***B.*** Migration was measured using a wound-healing assay. A wound was performed on MDA-MB-231 confluent cells seeded in 24 well plates. The medium was replaced by fresh medium containing 5 µM docetaxel in the absence of PAH, or the presence of 5 µM PAH. Two pictures were taken at two different points of each well immediately after wound, and 3 days after wound with 5X objective. The width of the wound was measured using ImageJ software and reported as final wound width/initial wound width in percentage. Data presented are the mean ± SEM of 3 independent experiments. Significance is attained at *P* < 0.05.

To study the effect of increasing chemotherapy efficacy by PTCH1 drug efflux inhibition on metastases formation, we performed migration assays. MDA-MB-231 and MDA-MB-468 cells were seeded on transwell membranes and treated with doxorubicin or docetaxel 24 or 48 h respectively in the absence or the presence of PAH. The observation on a microscope of the cells that migrated to the bottom of the wells after staining with crystal violet revealed that the addition of PAH at a concentration that has no effect alone strongly decreased the number of cells in the wells treated with doxorubicin or docetaxel (Fig. 7A). This suggests that the addition of PAH to the chemotherapies allowed to inhibit migration of cancer cells.

This conclusion was reinforced by wound-healing assays (Fig. 7B). Indeed, the addition of PAH to docetaxel treatment after wounding confluent MDA-MB-231 cells prevented wound closure to a greater extent than docetaxel alone, confirming that PTCH1 inhibition enables docetaxel to eliminate migrating cells. Note that PAH has no effect on wound closure by itself.

### PTCH1 contributes to drug efflux in TNBC cells

**Fig. 8:**
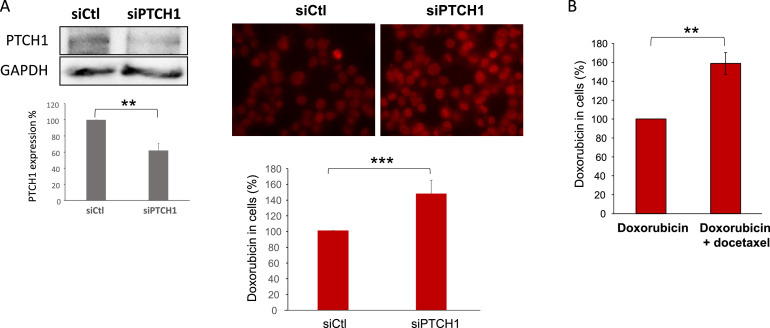
*PTCH1 contributes to the efflux of doxorubicin from TNBC cells.****A.*** PTCH1 protein expression (left panel) and intracellular doxorubicin (right panel) were analyzed 16 h after transfection of MDA-MB-231 cells with PTCH1-siRNA or negative-control-siRNA. PTCH1 and GAPDH western blot signals were quantified using ImageJ software (left panel). For doxorubicin accumulation measurements (right panel), MDA-MB-231 cells were grown on slides and transfected with PTCH1-siRNA or negative-control-siRNA. After incubation with 10 µM doxorubicin, 3 coverslips were fixed for doxorubicin loading control; the other coverslips were incubated with efflux buffer and fixed. **B.** Docetaxel inhibits the accumulation of doxorubicin in MDA-MB-231 cells. Cells on coverslip were incubated with 10 µM doxorubicin or 10 µM doxorubicin and 50 µM docetaxel. Doxorubicin fluorescence images were acquired by epifluorescence microscopy using a 40X objective, and doxorubicin fluorescence was quantified using ImageJ software for about 100 cells per condition per experiment. Histograms are the mean ± SEM of 3 independent experiments. Significance is attained at *P* < 0.05.

When the cells were treated with doxorubicin, which is naturally fluorescent, strong accumulation of the drug was observed in the cells (Fig. 8) as already described in [20]. Interestingly, the depletion of PTCH1 using specific silencing RNA in the MDA-MB-231 cell line increased intracellular accumulation of doxorubicin (Fig. 8A right panel). In this experiment, MDA-MB-231 cells were seeded on coverslips and transfected with 60 nM PTCH1-siRNA or negative-control-siRNA. PTCH1 protein expression (Fig. 8A left panel) and intracellular doxorubicin (Fig. 8A right panel) were analyzed 16 h after transfection. Western blots analyses showed that PTCH1-siRNA decreased PTCH1 protein expression by about 40%. After 1 h of incubation with doxorubicin, coverslips were fixed, and doxorubicin fluorescence was acquired and quantified, Results presented in Fig. 8A show that inhibiting PTCH1 expression leads to an increase in doxorubicin accumulation in MDA-MB-231 cells, and demonstrate that PTCH1 contributes to doxorubicin efflux in these cells.

We also observed that adding an excess of docetaxel to doxorubicin increased doxorubicin accumulation in MDA-MB-231 cells (Fig. 8B), indicating that docetaxel competes with doxorubicin for efflux via PTCH1. This is consistent with data showing that PTCH1 expression confers resistance to docetaxel in yeasts (Fig. 3) and confirms the role of PTCH1 in docetaxel efflux.

**Fig. 9:**
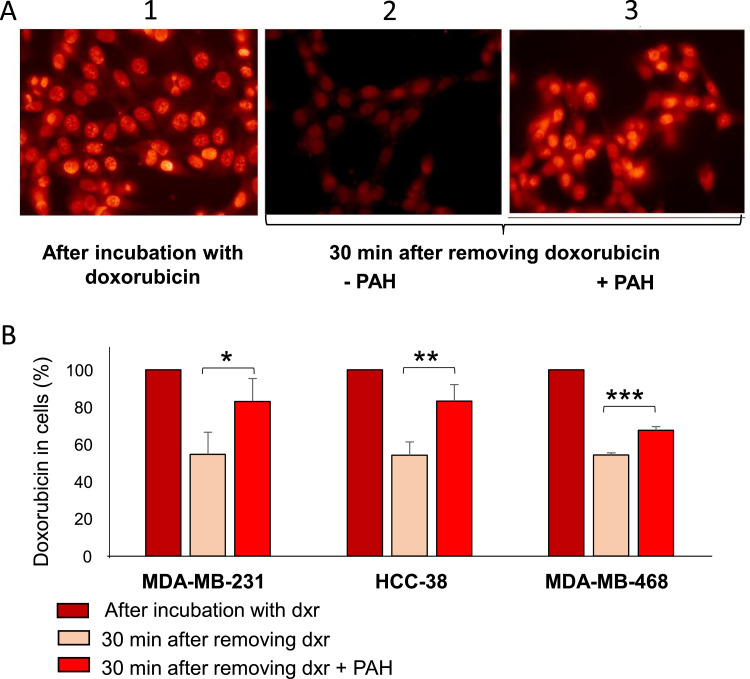
***PAH inhibits doxorubicin efflux from TNBC cells*. A.** Cells on coverslip were incubated 2 h with 10 µM doxorubicin, 3 coverslips were fixed, 6 coverslips were incubated with medium without doxorubicin ± PAH during 30 min and fixed. Fluorescence of doxorubicin in cells was then observed by epifluorescence microscopy. **B.** the doxorubicin fluorescence in cells was quantified using ImageJ software.

To further demonstrate that the increase in the efficacy of chemotherapy observed in TNBC cells is due to the inhibition of PTCH1 drug efflux activity, we quantified the amount of doxorubicin in cells that were or were not treated with PAH (Fig. 9). When the cells were treated with doxorubicin, strong accumulation of the drug was observed in the cells (Fig. 9A1). However, 30 min after doxorubicin was removed from the medium, the fluorescence intensity in the cells decreased significantly, indicating efflux of the drug from the cells (Fig. 9A2). Adding PAH to the efflux medium after removing doxorubicin enabled a relatively high level of fluorescence to be maintained in the cells, indicating that PAH inhibits doxorubicin efflux from cells (Fig. 9A3). Around 50% of doxorubicin was transported out of cells after 30 min in the absence of PAH, but this was significantly inhibited in the presence of PAH in the three TNBC cell lines (Fig. 9B). However, the effect of PAH is less pronounced on HCC-38 cells. This is consistent with the results of cell viability tests and suggests that other multidrug transporters may be involved in doxorubicin efflux from HCC-38 cells.

Taken together, our results imply that the enhanced cytotoxicity of doxorubicin and docetaxel observed in TNBC cells may be attributed to the inhibition of their efflux by PTCH1.

## Discussion

This study aimed to evaluate the role of PTCH1 in breast cancer cells' resistance to chemotherapy, and to assess the impact of PAH, an inhibitor of PTCH1 drug efflux activity, on the efficacy of chemotherapy treatments.

First, we analysed PTCH1 mRNA expression in tumour samples from breast cancer patient cohorts and observed PTCH1 mRNA expression in all breast tumour types, particularly in TNBC (Fig. 1A and 1B). Our data also revealed that PTCH1 mRNA was expressed in CTCs isolated from the blood of patients with metastatic breast cancer, particularly those with TNBC (Fig. 1C). Our analyses also showed that high PTCH1 mRNA expression is associated with a poor prognosis (Fig. 1D, Supplementary Fig. 1). Our observations are consistent with those reported in several studies [[16], [35], [36]]. Indeed, Wu et al. [36] found that PTCH1 is expressed in breast cancer tissue and that PTCH1 expression level is associated with a poor prognosis in breast cancer patients using the Oncomine database. Furthermore, analyses performed by Özcan and colleagues [35] on five GEO datasets and TCGA breast cancer data revealed that PTCH1 is a key predictor of resistance to neoadjuvant chemotherapy in oestrogen receptor-positive/human epidermal growth factor receptor 2-negative (ER+/HER2-) breast cancer.

TNBC is the most aggressive subtype of breast cancer. It is characterised by rapid disease progression, strong invasiveness and high recurrence and metastasis rates. It is also associated with a poor prognosis due to the limited treatment options available. Despite recent advances in targeted therapy and immunotherapy, chemotherapies such as platinum agents (cisplatin and carboplatin), taxanes (paclitaxel and docetaxel) and anthracyclines (doxorubicin and epirubicin) remain the standard treatment for TNBC at all stages [[33], [37], [38], [39], [40]]. However, the initial responses to these chemotherapies are mitigated by a high rate of chemoresistance and disease progression. A significant proportion of TNBC patients experience relapse within three to five years of treatment [[13], [41]]. Compared to other breast cancer subtypes, TNBC is enriched in a subpopulation of cells with self-renewal ability. These cells are termed breast cancer stem cells (BCSCs) or tumour-initiating cells (TICs), and are thought to be drug-resistant and involved in the high relapse rate of TNBC patients [42]. As PTCH1 is expressed in TNBC patients and TNBC cell lines, and as we have previously demonstrated its involvement in the chemotherapy resistance of melanoma and adrenocortical carcinoma cells [19,20], we investigated its potential role in drug resistance in TNBC cells.

Previous studies have demonstrated that PTCH1 can efflux doxorubicin and that PAH can inhibit this process in yeasts overexpressing PTCH1 and in melanoma cell lines expressing endogenous PTCH1 [[17], [20], [23]]. In the present work, we observed that inhibiting PTCH1 expression using silencing RNA specific of PTCH1 enhanced doxorubicin accumulation in one of the TNBC cell lines that express PTCH1 endogenously, suggesting that PTCH1 contributes to the efflux of doxorubicin from these cells (Fig. 8A), and that PAH inhibits doxorubicin efflux from three TNBC cell lines that express PTCH1 endogenously (MDA-MB-231, HCC-38 and MDA-MB-468) (see Fig. 9). This demonstrates that PTCH1 is involved in doxorubicin efflux in TNBC cell lines and that PAH can also inhibit the doxorubicin efflux activity of PTCH1 in these cells. The observation that an excess of docetaxel inhibited doxorubicin efflux from a TNBC cell line suggests that docetaxel uses the same efflux pathway as doxorubicin (Fig. 8B). We also demonstrated that PTCH1 expression in yeast conferred docetaxel resistance, which was inhibited by the addition of PAH to the culture medium (see Fig. 3B). These results demonstrate that PTCH1 can efflux docetaxel, thereby inducing resistance to this chemotherapeutic agent.

Our data revealed that PAH significantly enhanced the cytotoxic effect of doxorubicin and docetaxel on three TNBC cell lines grown in 2D (Fig. 4) and 3D (spheroids) (Fig. 6). This *in vitro* model more closely resembles a tumour than adherent cells. Notably, the cell line with the highest PTCH1 expression (HCC-38) exhibited the strongest resistance to these two chemotherapies (see Figs. 2B, 4 and Supplementary Table 1). However, PAH had a relatively low effect on the efficacy of doxorubicin and docetaxel in HCC-38 compared to MDA-MB-231 and MDA-MB-468. We found that HCC-38 expressed higher amounts of P-glycoprotein (P-gp) and ABCG2 than MDA-MB-231 and MDA-MB-468 (see Fig. 5). These two multidrug transporters, which belong to the ABC transporter family, are known to trigger resistance to doxorubicin and docetaxel [[43], [44], [45]]. Despite its high expression in HCC-38, PTCH1 probably contributes less to chemotherapy resistance than P-gp and ABCG2 do in HCC-38 compared to MDA-MB-231 and MDA-MB-468. Furthermore, we observed that adding PAH to docetaxel prevented TNBC cell migration (Fig. 7). As PTCH1 is over-expressed in subpopulations of cancer stem cells with migratory properties, inhibiting its drug efflux activity with PAH made docetaxel toxic to these cells, allowing the elimination of migratory cells.

Taken together, our data suggest that PTCH1 is involved in the resistance of TNBC cells to chemotherapy, and that the use of an inhibitor of PTCH1 drug efflux during neoadjuvant or adjuvant therapy could enhance the efficacy of the treatment against TNBC expressing PTCH1, and prevent resistance of TNBC cells to treatment, relapse and metastases formation. Our study highlights the therapeutic potential of targeting PTCH1 using drug association strategies for the treatment of TNBC patients.

## Funding

This work was supported by grants from Centre National de la Recherche Scientifique (CNRS), Association France Cancer, LABEX SIGNALIFE (ANR-11-LABX-0028) and IDEX UCA Jedi (ANR-15-IDEX-01) from the program Investments for the Future of the French National Agency for Research.

## CRediT authorship contribution statement

**Sarah Cogoluegnes:** Investigation, Formal analysis, Data curation. **Sandra Kovachka:** Writing – review & editing, Methodology, Investigation, Formal analysis, Data curation, Conceptualization. **Thierry Dubois:** Writing – review & editing, Writing – original draft, Supervision, Methodology, Investigation, Formal analysis, Data curation, Conceptualization. **Roberto Würth:** Writing – review & editing, Formal analysis, Data curation. **Elisa Donato:** Methodology, Formal analysis, Data curation. **Andreas Trumpp:** Methodology, Formal analysis, Data curation. **Michel Franco:** Writing – review & editing, Resources. **Frédéric Luton:** Writing – review & editing, Resources. **Stéphane Azoulay:** Writing – review & editing, Supervision, Funding acquisition, Conceptualization. **Isabelle Mus-Veteau:** Writing – original draft, Validation, Supervision, Resources, Project administration, Methodology, Investigation, Funding acquisition, Formal analysis, Conceptualization.

## Declaration of competing interest

The authors declare no conflict of interest.

## Appendix Supplementary materials

Image, application 1
