# Supplementary material for: Inhibition of PTCH1 drug efflux activity enhances chemotherapy efficacy against triple negative breast cancers
Source: Transl Oncol. 2026 Apr 18;68:102777. doi: 10.1016/j.tranon.2026.102777 (PMC13101699; doi:10.1016/j.tranon.2026.102777)
Supplement: Supplementary file 1 [file mmc1.docx]

**Supplementary data**

***Sup Figure 1. High PTCH1 mRNA expression is associated with a poorer prognosis in breast cancer.*** Distant Metastasis Free Survival (DMFS), disease-free survival (DFS) and overall survival (OS) data based on *PTCH1* mRNA expression were obtained from the intrinsic molecular subtypes’ prognostic analysis on ER+/HER2- high proliferative breast cancers performed on bc-GenExMiner v4.5 web portal and illustrated by Kaplan–Meier curves. The obtained Hazard Ratio (HR) with 95% confidence interval and log-rank P-values are shown.

|  |  | *MDA-MB-231* | | *MDA-MB-468* | | *HCC-38* | |
| --- | --- | --- | --- | --- | --- | --- | --- |
|  | **PAH (µM)** | **0** | **15** | **0** | **15** | **0** | **15** |
| *Doxorubicin* | IC50 (µM) | 16.6μ2 | 2.3μ0.3 | 33.6μ1.4 | 4.8μ2 | 47.8μ12 | 12μ0.8 |
|  | Efficacy |  | x 7 |  | x 7 |  | x 4 |
| *Docetaxel* | IC50 (µM) | 93.6μ8 | 32.6μ7 | 158.6μ5 | 92μ13 | 178.2μ21 | 144.8μ16 |
|  | Efficacy |  | x 2.9 |  | x 1.7 |  | x 1.2 |

***Sup Table 1. PTCH1 drug efflux inhibitor PAH increases the cytotoxicity of docetaxel and doxorubicin against TNBC cells****.* Cell viability was measured after 24 hours or 48 hours treatment with increasing concentration of doxorubicin or docetaxel respectively on MDA-MB-231, MDA-MB-468 and HCC-38 cell lines in the absence or the presence of 15µM PAH. IC_50_ values of chemotherapy (corresponding to the concentration of chemotherapy inducing 50% of cell death) were calculated. Data reported are the mean ± SEM of at least 3 independent experiments.
